# Subgingival microflora in adolescent females with polycystic ovary syndrome and its association with oral hygiene, gingivitis, and selected metabolic and hormonal parameters

**DOI:** 10.1007/s00784-020-03456-5

**Published:** 2020-08-10

**Authors:** Natalia Wendland, Justyna Opydo-Szymaczek, Małgorzata Mizgier, Grażyna Jarząbek-Bielecka

**Affiliations:** 1 Department of Pediatric Dentistry, Chair of Pediatric Dentistry, Poznan University of Medical Sciences, 70 Bukowska Street, 60-812 Poznan, Poland; 2 Department of Dietetics, Faculty of Physical Culture in Gorzow Wielkopolski, Poznan University of Physical Education, 4-6 Orląt Lwowskich Street, 66-400 Gorzów Wielkopolski, Poland; 3 Department of Perinatology and Gynecology, Division of Developmental Gynecology and Sexology, Poznan University of Medical Sciences, 22 Polna Street, 60-535 Poznan, Poland

**Keywords:** Polycystic ovary syndrome, Gingivitis, Subgingival microflora, Lipid profile, Insulin resistance

## Abstract

**Objectives:**

Research studies suggest that polycystic ovary syndrome (PCOS) may influence the composition of the oral microflora in women. This study aimed to investigate factors affecting the number of selected periopathogens in a young cohort of females with PCOS and to assess the association between oral hygiene, subgingival microbiome, gingival health, and metabolic and hormonal parameters.

**Materials and methods:**

Thirty-two subjects with PCOS and twenty-three healthy controls aged 15–19 years were examined periodontally by a calibrated dentist. A real-time PCR method was used for the identification of 9 subgingival microorganisms. Subjects with PCOS underwent blood tests for determination of FSH, LH, total testosterone, DHEA-S, estradiol, SHBG, fasting glucose, fasting insulin, and lipid profile.

**Results:**

Gingival index (GI), the proportion of bleeding sites (BOP%), probing depth (PD), and plaque index (PLI) did not differ significantly between cases and healthy age-mates. The control group had significantly higher levels of *Peptostreptococcus micros* and substantially greater percentage of subjects infected by *Treponema denticola. Capnocytophaga gingivalis* count was positively correlated with the level of estradiol, while the concentration of HDL-C was negatively correlated with the number of *Aggregatibacter actinomycetemcomitans* and orange complex bacteria.

**Conclusions:**

PCOS in young patients was not associated with higher pathogenicity of subgingival biofilms.

**Clinical relevance:**

Further studies are needed to explain the relationship between hormonal and metabolic abnormalities, subgingival microflora, and periodontal health in patients with PCOS.

## Introduction

Dental plaque is the primary etiologic factor for the initiation of periodontal disease. However, variations in sex hormones may modify the susceptibility of the host, increasing the periodontal inflammatory response to periodontal pathogens [1, 2]. Current periodontal disease classification recognizes the impact of sex hormones on the periodontium. Under the category of dental plaque–induced gingival diseases mediated by systemic risk factors, those associated with puberty, menstrual cycle, pregnancy, and oral contraceptives are classified as gingivitis exacerbated by sex steroid hormones [2].

Recent studies also showed a significant association between periodontal disease and polycystic ovary syndrome (PCOS) [3–9]. PCOS is the most common endocrinopathy, affecting women of reproductive age with the prevalence ranging from 15 to 20% according to the Rotterdam criteria [10, 11]. It is a complex disease with characteristics of hyperandrogenism and chronic anovulation. Women with PCOS have an adverse cardiometabolic risk profile, including insulin resistance, visceral obesity, and dyslipidemia [10]. The mechanisms that link periodontal disease and PCOS are not entirely understood. Some research studies suggest that PCOS may affect the composition of the oral microflora [5, 12] and modify the systemic antibody responses to selective members of this microbial community [5].

To our knowledge, there are no studies evaluating the presence of periopathogens in adolescent girls with PCOS who have periodontal disease at the gingival level without loss of the connective tissue attachment. The diagnosis of periodontal problems and evaluation of risk factors in adolescence is important, because the incipient forms of periodontal disease, such as gingival inflammation, may progress to advanced irreversible periodontitis in adults.

Thus, this study aimed to investigate factors affecting the level of selected periopathogens in a young cohort of females with PCOS and to assess the association between oral hygiene, subgingival microbiome, gingival health, and metabolic and hormonal parameters.

## Materials and methods

The study was approved by the Bioethics Committee at the Poznan University of Medical Sciences (resolution no. 536/18), and written informed consent was obtained from the parents and patients.

### Study population

The study group comprised 32 Caucasian females aged 15–19 years (mean ± SD = 16.4 ± 1.1). They were recruited among patients admitted to the Gynecology and Perinatology Medical Clinic at the Gynecology and Obstetrics Hospital of Poznan University of Medical Sciences (Poznan, Wielkopolska province). All subjects were at least 2 years after menarche and sought medical diagnosis for the signs of hormonal abnormalities. Inclusion to the PCOS group was based on the 2003 Rotterdam criteria [10, 11], with the presence of at least two of the following:

- clinical and/or biochemical hyperandrogenism (hirsutism with moderate to severe acne, and/or the elevation of total testosterone or free testosterone levels),
- oligoovulation (based on oligomenorrhea defined as bleeding episodes occurring less than 8 times per year or secondary amenorrhea),
- polycystic ovaries (at least 12 follicles in each ovary each measuring 2–9 mm in diameter and/or ovarian volume > 10 ml) on ultrasound.

The exclusion criteria were as follows: any systemic disease or medications of continuous use, hormonal therapy, Cushing syndrome, congenital adrenal hyperplasia, hyperprolactinemia suggestive of pituitary adenoma, thyroid dysfunction, diabetes, androgen-secreting tumors, the use of orthodontic appliances, the presence of untreated non-vital teeth with open pulp chambers or clinical signs of periapical inflammation, smoking, antibacterial mouthwashes, or the use of antibiotics during the past 6 months.

The control group of 23 healthy females aged 15–19 (mean ± SD = 16.5 ± 1.5) was recruited among patients visiting the University Center of Stomatology and Specialist Medicine in Poznan for a routine check-up. The control group was matched with regard to age and oral hygiene to the PCOS group.

The exclusion criteria were as follows: any systemic disease or medications of continuous use, hormonal therapy, signs of possible hormonal disturbances such as menstrual disorders (amenorrhea, oligomenorrhea, irregular periods, short or heavy menstrual bleedings), obesity, moderate to severe acne according to Investigator’s Global Assessment scale, hirsutism (> 7 points on the modified Ferriman-Gallwey scale) [13–15], the presence of untreated non-vital teeth with open pulp chambers or clinical signs of periapical inflammation, smoking, poor oral hygiene, the use of orthodontic appliances, antibacterial mouthwashes, or the use of antibiotics during the past 6 months.

### Medical evaluation

After admission to the hospital, patients from the study group had basic anthropometric measurements, including height and weight. Diagnosis and classification of overweight and obesity were based on body mass index (BMI), calculated as a weight in kilograms divided by the square of a height in meters (kg/m^2^). According to WHO, for children aged 5–19 years, overweight and obesity correspond to BMI-for-age greater than 1 standard deviation and 2 standard deviations above the WHO growth reference median, respectively [16].

Blood samplings and gynecological examination with ultrasound were performed in the early follicular phase (days 3–5), apart from patients with secondary amenorrhea.

Hormonal and biochemical parameters, including FSH, LH, total testosterone, estradiol, DHEA-S, SHBG, fasting glucose and insulin, total cholesterol (TC), high-density lipoprotein cholesterol (HDL-C), and triglycerides (TG), were measured in the morning after overnight fasting.

After 2 days of hospital stay, the participants were referred to the University Center of Stomatology and Specialist Medicine, where they were clinically evaluated by a one calibrated dentist (N.W.).

The control group of patients, recruited initially in the University Center of Stomatology and Specialist Medicine based on oral hygiene and oral health screening, was referred for the gynecological consultation in the hospital outpatient department. The examiner (G.J.B.) verified the presence of any symptoms of hormonal disturbances in the control group. Participants answered questions about menstrual cycle regularity*.* Physical examination included the following: measurement of weight and height, evaluation of skin for signs of virilization, such as acne and hirsutism. Due to ethical concerns about unnecessary invasive procedures in minors, ultrasound examination and blood tests were carried out only in the study group. Subjects from the control group who fulfilled inclusion criteria were further examined in the University Center of Stomatology and Specialist Medicine.

### Dental examination

The measurement of the state of oral hygiene by the Silness-Löe plaque index (PLI) was based on recording both soft debris and mineralized deposits on the six index teeth: 16, 12, 24, 36, 32, 44. Each of the four surfaces of the teeth (buccal, lingual, mesial, and distal) was given a score from 0 to 3. The scores from the four areas of the tooth were added and divided by four to give the plaque index for the tooth with the following scores and criteria: 0—no plaque; 1—a film of plaque adhering to the free gingival margin and adjacent area of the tooth detected by the probe; 2—moderate accumulation of soft deposits within the gingival pocket, or the tooth and gingival margin which can be seen with the naked eye; 3—abundance of soft matter within the gingival pocket and/or on the tooth and gingival margin. The subject’s plaque status was assigned as follows: good (0–0.6), average (0.7–1.8), and poor (1.9–3.0) [17].

Four permanent teeth (maxillary right first molar, maxillary right central incisor, mandibular left first molar, and mandibular left central incisor) were chosen for subgingival sampling. Diagnostic kit PET Test® plus (MIP Pharma, Germany) was used to assess the presence and quantity of 9 periodontal pathogens: *Aggregatibacter actinomycetemcomitans* (*A. actinomycetemcomitans*), *Porphyromonas gingivalis* (*P. gingivalis*), *Tannerella forsythia* (*T. forsythia*), *Treponema denticola* (*T. denticola*), *Fusobacterium nucleatum* (*F. nucleatum*), *Prevotella intermedia* (*P. intermedia*)*, Peptostreptococcus micros* (*P. micros*), *Eubacterium nodatum* (*E. nodatum*), and *Capnocytophaga gingivalis* (*C. gingivalis*), as well as total subgingival bacterial count. Four samples were collected from each patient according to the manufacturer’s instruction. Before inserting paper points into buccal sites of gingival sulci, the supragingival bacterial plaque was cleaned with the sterile cotton pellets (taking care not to induce bleeding), and the examined sites were dried and isolated from saliva with sterile swabs. Sterile paper points from the diagnostic kit were inserted to the full depth of the gingival crevice for 20 s, with the use of sterile tweezers. Then each of the four samples collected from the patient was loaded into one test tube to form one collective specimen, placed in a transportation set, and shipped to a MIP Pharma Laboratory. Sample analysis was performed by using a real-time polymerase chain reaction (PCR). Free strand sections of DNA were obtained from lysed bacterial cells and were subsequently subjected to amplification and hybridization with fluorescence-stained starters. Quantitative analysis was performed with a reader that measures the intensity of fluorescence compared with that in reference specimens [18].

The gingival condition was assessed with the use of the gingival index (GI) by Löe and Silness on the six index teeth: 16, 12, 24, 36, 32, 44. It scores the marginal and interproximal tissues separately. The criteria were as follows: 0 = normal gingiva; 1 = mild inflammation—slight change in color and slight edema but no bleeding on probing; 2 = moderate inflammation—redness, edema, and glazing, bleeding on probing; 3 = severe inflammation—marked redness and edema, ulceration with a tendency to spontaneous bleeding. The scores of the four areas of the tooth were summed and divided by four to give the GI for the tooth. The GI of the individual was obtained by adding the values of each index tooth and dividing by the number of teeth examined. The mean GI was used to determine categorical gingival status as follows: 0, healthy; 0.1–1, mild gingivitis; 1.1–2, moderate gingivitis; 2.1–3, severe gingivitis [17, 19].

Probing depth (PD) was defined as the distance between the gingival margin and the bottom of the sulcus. The PD measurements were done with the use of a Williams manual periodontal probe (Hu-Friedy Mfg. Co. LLC, Chicago, USA) which has millimeter markings at 1, 2, 3, 5, 7, 8, 9, and 10 mm. When the PD measurement was between 2 marks on the probe, it was rounded off to the closest 0.5 mm. Bleeding upon probing score (BOP%) was assessed as the proportion of bleeding sites when stimulated by a periodontal probe. The measurements were recorded after collection of subgingival plaque samples on all teeth present at six sites (distobuccal, buccal, mesiobuccal, distolingual, lingual, and mesiolingual) [2].

All subjects were examined by a single dentist (N.W.), who was calibrated before the start of this study by the experienced dental practitioner (J.O.S.). Calibration exercises were performed on adolescent patients, apart from the main study. Participants were evaluated by each examiner, and N.W. was considered calibrated when her measurements reached a substantial correlation with J.O.S. evaluations, as well as considerable correlation of repeated assessments (Cohen’s Kappa > 80).

### Biochemical parameters

Biochemical analyses were performed in the central hospital laboratory. Plasma glucose was measured by the enzymatic method with hexokinase. Insulin, follicle-stimulating hormone (FSH), luteinizing hormone (LH), total testosterone, 17-β-estradiol, dehydroepiandrosterone sulfate (DHEA-S), and sex hormone–binding globulin (SHBG) were measured by electrochemiluminescence (ECLIA) immunoassay method (Elecsys) (Roche Diagnostics Gmbh, Mannheim, Germany). Plasma total cholesterol (TC), high-density lipoprotein cholesterol (HDL-C), and triglycerides (TG) levels were determined by the enzymatic colorimetric method (Roche Diagnostics GmbH, Mannheim, Germany). Low-density lipoprotein cholesterol (LDL-C) was calculated with the use of Friedewald’s formula (LDL-C [mg/dl] = TC − HDL − C − TG/5). The free testosterone was calculated from total testosterone and SHBG levels using the online free testosterone calculator available on the ISSAM website (www.issam.ch/freetesto.htm). Homeostasis model assessment of insulin resistance (HOMA-IR) was applied using the following formula: HOMA-IR = fasting insulin (μU/mL) × fasting glucose (mmol)/22.5 [20, 21]. Results were compared with the age- and sex-specific laboratory reference ranges and the literature data [20–22].

### Statistical analysis

Based on the previous reports on a periodontal status of young females suffering from PCOS [7, 8], a difference of 0.50 in the gingival index (GI) and SD of 0.6 were considered as reference values. Power calculation was performed with the G*Power 3.1.9.2 software. This analysis indicated that with 20 subjects in every group, the study would have 80% power to detect the anticipated difference in the GI between individuals with PCOS and healthy controls, with alfa set at 0.05.

Data analysis was performed using the Statistica software version 12 (StatSoft. Inc. 2014, Tulsa, USA) with significance taken as *p* < 0.05. All parameters were given as mean ± SD (standard deviation), median, and range. Since many variables were not normally distributed, Spearman’s rank test was used for analyses of correlation between quantitative data, the Mann-Whitney *U* test for the evaluation of differences between numerical data, and chi-square tests to compare the categorical variables. The Yates correction for continuity was used when the observed frequency was small (< 5).

We did not use the Bonferroni correction to control for multiple comparisons, because our study was explanatory in nature and a conservative correction would increase the probability of a type II error.

## Results

Table 1 presents characteristics of the subjects including age, selected anthropometric, hormonal, biochemical, and clinical parameters, as well as reference values according to the hospital laboratory and literature data. The mean age of the study group and the control group was similar (16.4 and 16.5, respectively). BMI index of subjects from the study group was significantly higher as compared with the BMI index of the control group (25.1 and 20.5, respectively). There were 8 subjects with obesity and 7 with overweight in the study group, while in the control group, only 3 subjects had BMI index above 1SD of the growth reference median. PLI, GI, BOP%, and PD of both groups did not differ significantly (*p* > 0.05). There were 6 patients with moderate gingivitis: 3 in the study group and 3 among healthy controls. Gingivitis defined as ≥ 10% of sites with bleeding on probing was noted in 18 (56.2%) of the subjects with PCOS and 13(56.5%) of the control age-mates.

**Table 1:** Characteristics of the examined subjects including age, selected anthropometric, hormonal, biochemical and clinical parameters

	No. of subjects with values determined	Mean	Median	Min	Max	SD	Reference value	No. of subjects with abnormal values
Age of the study group	32	16.4^1^	16	15	19	1.1	N.A. (not applicable)	N.A.
Age of the control group	23	16.5	16	15	19	1.5		
BMI of the study group	32	25.08^2^	24.08	16.00	42.99	5.90	< 1 SD of the reference median [16]	15↑
BMI of the control group	23	20.45	20.05	16.20	26.70	2.93		3↑
PLI of the study group	32	0.62^3^	0.58	0.08	1.42	0.37	≤ 0.6 (good hygiene) [19]	↑13
PLI of the control group	23	0.73	0.83	0.04	1.54	0.47		↑14
GI of the study group	32	0.47^4^	0.42	0.08	1.29	0.34	≤ 1 mild gingivitis [17]	↑3
GI of the control group	23	0.47	0.38	0.00	1.33	0.39		↑3
BOP% of the study group	32	13.2^5^	11.7	1.6	38.7	9.3	< 10% gingival health [23]	↑18
BOP% of the control group	23	15.7	12.1	1.2	45.3	12.6		↑13
PD of the study group	32	0.85^6^	0.86	0.50	1.00	0.11	≤ 3 [23]	0
PD of the control group	23	0.80	0.83	0.50	1.00	0.16		0
FSH (mlU/ml)	32	5.07	5.33	1.32	9.42	2.05	3.5–12.5*	7 ↓
LH (mlU/ml)	32	11.91	9.70	0.20	27.90	7.77	2.4–12.6*	14↑, 2↓
LH/FSH	32	2.34	2.24	0.15	8.54	1.60	≤ 2 [22]	17↑
Estradiol (pg/ml)	32	59.83	44.18	12.55	236.40	52.80	12.5–166* (follicular phase)	3↑
Total testosterone (ng/dl)	32	51.16	49.50	6.00	88.00	17.58	6–82*	1↑
SHBG (nmol/l)	32	46.87	43.17	13.86	109.80	25.88	26.1–110*	8 ↓
Free testosterone (ng/l)	32	8.36	7.35	1.07	17.60	4.30	≤ 9.5 [20]	19 ↑
DHEA-S (μmol/l)	32	7.65	7.12	2.17	12.80	2.98	1.77–9.99*	9↑
Fasting glucose (mg/dl)	32	87.61	87.45	77.20	99.00	5.77	60–99 *	0
Fasting insulin (μU/ml)	32	15.30	14.71	5.45	31.51	6.35	2.6–24.9*	4↑
HOMA-IR	32	3.41	3.29	1.06	7.05	1.36	< 2.32 [21]	26↑
TC (mg/dl)	32	155.52	160.30	120.50	207.70	22.17	< 190*	1↑
HDL-C (mg/dl)	32	54.06	52.75	38.30	73.20	8.65	≥ 45*	3↓
LDL-C (mg/dl)	32	82.71	82.45	44.30	130.60	19.52	< 115*	1↑
TG (mg/dl)	32	93.74	86.45	46.70	176.80	36.32	< 150*	4↑

*BMI* body mass index, *PLI* plaque index, *GI* gingival index, *BOP%* bleeding on probing score, *PD* probing depth, *FSH* follicle-stimulating hormone, *LH* luteinizing hormone, *SHGB* sex hormone–binding globulin, *DHEA-S* dehydroepiandrosterone sulfate, *HOMA-IR* homeostatic model assessment of insulin resistance, *TC* total cholesterol, *HDL-C* high-density lipoprotein cholesterol, *LDL-C* low-density lipoprotein cholesterol, *TG* triglycerides

*According to the hospital age- and sex-specific laboratory reference ranges, ↑ above, ↓ below the reference values

^1^*p* = 0.8047 as compared with the age of the control group, Mann-Whitney *U* test, not significant

^2^*p* = 0.0014 as compared with the BMI of the control group, Mann-Whitney *U* test, statistically significant

^3^*p* = 0.3884 as compared with PLI of the control group, Mann-Whitney *U* test, not significant

^4^*p* = 0.8710 as compared with GI of the control group, Mann-Whitney *U* test, not significant

^5^*p* = 0.6696 as compared with BOP% of the control group, Mann-Whitney *U* test, not significant

^6^*p* = 0.2653 as compared with PD of the control group, Mann-Whitney U test, not significant

The periodontal pathogens tested were expressed at various levels in the study and in the control group (Table 2). *C. gingivalis*, *P. intermedia*, and *P. gingivalis* were the most abundant species in the study group, while the control group showed the highest count of *P. intermedia*, then *C. gingivalis*, and no *P. gingivalis*. *E. nodatum* from yellow-orange-associated complex was not detected in the subjects of the study. From the detectable species, the least abundant was *A*. *actinomycetemcomitans. T. denticola*, *P. micros*, and orange complex bacteria were significantly more often isolated in the control group. Subjects with moderate gingivitis had significantly higher levels of all species from red complex and *P. micros*, and significantly higher percentage of subjects infected by red complex bacteria and *T. denticola*, as compared with subjects with healthy gingiva/mild gingivitis (*p* < 0.05).

**Table 2:** Comparison of the number of bacteria isolated from gingival sulci and the percentage of patients infected by particular pathogens in the study group and the control group of subjects, in the subgroups with moderate gingivitis and healthy gingiva/mild gingivitis

	Subjects with PCOS		Control group		Mann-Whitney U test	Chi-square test	GI ≤ 1healthy gingiva/mild gingivitis *n* = 49		GI > 1moderate gingivitis *n* = 6		Mann-Whitney U test	Chi-square test
Periopathogens tested	Mean ± SD	No (%) of infected patients	Mean ± SD	No (%) of infected patients	p	p	Mean ± SD	No (%) of infected patients	Mean ± SD	No (%) of infected patients	p	p
*A. actinomycetemcomitans*	5.5 × 10 ± 20.8 × 10	3 (9.4%)	1.9 × 10 ± 9.0 × 10	1 (4.3%)	0.5072	0. 8557	4.5 × 10 ± 17.8 × 10	4 (8.2%)	0 ± 0	0 (0%)	0.4905	0.9156
Red complex bacteria	2.1 × 10^4^ ± 15.0 × 10^4^	10 (31.3%)	6.7 × 10^3^ ± 20.8 × 10^3^	15 (65.2%)	0.0990	0.0126*	4.5 × 10^3^ ± 15.0 × 10^3^	19 (38.8%)	9.8 × 10^4^ ± 22 × 10^4^	6 (100%)	0.0019*	0.0160*
*P. gingivalis*	1.6 × 10^4^ ± 8.8 × 10^4^	4 (12.5%)	0 ± 0	0 (0%)	0.0847	0.2170	2.0 × 10^2^ ± 11.0 × 10^2^	2 (4.1%)	8.4 × 10^4^ ± 20.4 × 10^4^	2 (33.3%)	0.0108*	0.0765
*T. forsythia*	2.3 × 10^3^ ± 7.0 × 10^3^	6 (18.8%)	8.7 × 10^2^ ± 22.5 × 10^2^	8 (34.8%)	0.3434	0.1782	1.2 × 10^3^ ± 4.1 × 10^3^	10 (20.4%)	6.2 × 10^3^ ± 11.9 × 10^3^	4 (66.7%)	0.0190*	0.0501
*T. denticola*	2.2 × 10^3^ ± 5.2 × 10^3^	9 (28.1%)	5.8 × 10^3^ ± 19.0 × 10^3^	14 (60.9%)	0.0521	0.0152*	3.1 × 10^3^ ± 13.4 × 10^3^	17 (34.7%)	8.4 × 10^3^ ± 6.9 × 10^3^	6 (100%)	0.0005*	0.0087*
Orange complex bacteria	3.4 × 10^4^ ± 15.0 × 10^4^	16 (50.0%)	5.0 × 10^4^ ± 15.8 × 10^4^	20 (87.0%)	0.0610	0.0045*	1.8 × 10^4^ ± 8.8 × 10^4^	31 (63.3%)	22.3 × 10^4^ ± 36.2 × 10^4^	5 (83.3%)	0.1680	0.6024
*F. nucleatum*	0.9 × 10^3^ ± 2.6 × 10^3^	11 (34.4%)	3.2 × 10^2^ ± 10.0 × 10^2^	6 (26.1%)	0.4786	0.5118	6.9 × 10^2^ ± 22.0 × 10^2^	16 (32.7%)	1.4 × 10^2^ ± 3.4 × 10^2^	1 (16.7%)	0.4886	0.7400
*P. intermedia*	3.3 × 10^4^ ± 15.0 × 10^4^	6 (18.8%)	4.8 × 10^4^ ± 15.6 × 10^4^	5 (21.7%)	0.7976	0.7846	1.7 × 10^4^ ± 8.6 × 10^4^	9 (18.4%)	2.2 × 10^5^ ± 3.6 × 10^5^	2 (33.3%)	0.2543	0.7456
*P. micros*	0.6 × 10^3^ ± 1.3 × 10^3^	13 (40.6%)	1.3 × 10^3^ ± 2.5 × 10^3^	17 (73.9%)	0.0428*	0.0145*	7.9 × 10^2^ ± 19.7 × 10^2^	25 (51.0%)	1.5 × 10^3^ ± 1.4 × 10^3^	5 (83.3%)	0.0358*	0.2864
*E. nodatum*	0 ± 0	0 (0%)	0 ± 0	0 (0%)	N.A.	N.A.	0 ± 0	0 (0%)	0 ± 0	0 (0%)	N.A.	N.A.
*C. gingivalis*	5.9 × 10^4^ ± 6.8 × 10^4^	32 (100%)	3.1 × 10^4^ ± 4.3 × 10^4^	23 (100%)	0.0605	N.A.	4.5 × 10^4^ ± 4.8 × 10^4^	49 (100%)	6.4 × 10^4^ ± 12.6 × 10^4^	6 (100%)	0.4660	N.A.
total bacteria count	4.7 × 10^8^ ± 12.6 × 10^8^	32 (100%)	3.9 × 10^8^ ± 8.1 × 10^8^	23 (100%)	0.3018	N.A.	4.7 × 10^8^ ± 11.5 × 10^8^	49 (100%)	1.1 × 10^8^ ± 0.8 × 10^8^	6 (100%)	0.3950	N.A.

*p < 0.05, statistically significant differences between groups

Among the dental plaque–related variables, PLI was significantly correlated with GI and BOP% (*p* < 0.0001), while BOP% was significantly correlated with *T. denticola* and *T. forsythia* counts, and with the number of bacteria from red complex. PD was not significantly correlated with any plaque-related variables (Table 3).

**Table 3:** Spearmann correlation coefficients between GI, BOP%, and PD and dental plaque–related variables

	GI		BOP%		PD	
	Correlation coefficient	*p*	Correlation coefficient	*p*	Correlation coefficient	*p*
PLI	0.68	0.0000*	0.78	0.0000*	0.24	0.0746
Number of *A. actinomycetemcomitans*	− 0.01	0.9416	− 0.13	0.3612	− 0.18	0.1973
Number of bacteria from red complex	0.14	0.3254	0.29	0.0332*	0.22	0.1044
Number of *P. gingivalis*	0.01	0.9143	0.02	0.9048	0.13	0.3301
Number of *T. forsythia*	0.17	0.2130	0.30	0.0237*	0.17	0.2283
Number of *T. denticola*	0.19	0.1691	0.33	0.0148*	0.25	0.0670
Number of bacteria from orange complex	0.14	0.2911	0.24	0.0839	0.02	0.8811
Number of *F. nucleatum*	0.09	0.5220	0.17	0.2156	0.02	0.8682
Number of *P. micros*	− 0.06	0.6858	0.03	0.8548	0.00	0.9898
Number of *P. intermedia*	0.20	0.1456	0.26	0.0532	0.23	0.0871
Number of *C. gingivalis* (green complex)	− 0.14	0.3091	− 0.08	0.5541	− 0.01	0.9333
Total bacteria count	0.05	0.7166	0.17	0.2278	− 0.13	0.3258

*Statistically significant correlations (*p* < 0.05)

Table 4 presents the Spearman correlation coefficients for pairwise variables and *p* values (*p*) of statistically significant associations. Out of 165 correlations, only 6 reached the level statistical significance. PLI was significantly correlated with the count of red complex bacteria, orange complex bacteria, *T. denticola*, and *F. nucleatum* (*p* = 0.0497, *p* = 0.0145, *p* = 0.0154, and *p* = 0.0380, respectively). The number of *C. gingivalis* was positively correlated with the level of estradiol (*p* = 0.0478), while the concentration of HDL-C was negatively correlated with the count of *A. actinomycetemcomitans* and orange complex bacteria (*p* = 0.0283 and *p* = 0.0452, respectively). All correlations were weak (correlation coefficients below 0.4/above − 0.4).

**Table 4:** Spearmann correlation coefficients between hormonal and metabolic parameters and counts of the bacterial species

	No of subjects	*A. actinomycetemcomitans*	Red complex bacteria	*P. gingivalis*	*T. forsythia*	*T. denticola*	Orange complex bacteria	*F. nucleatum*	*P. micros*	*P. intermedia*	*C. gingivalis*	Total bacteria count
Age	55	− 0.05	0.02	− 0.16	0.10	0.06	0.02	− 0.25	− 0.08	0.19	0.15	− 0.03
BMI	55	0.05	− 0.14	− 0.07	− 0.12	− 0.10	0.03	− 0.03	− 0.06	− 0.06	0.09	− 0.05
Estradiol (pg/ml)	32	− 0.28	− 0.05	0.00	− 0.06	− 0.08	0.32	0.21	0.31	0.24	0.35^7^	0.00
Total testosterone (ng/dl)	32	− 0.17	− 0.08	− 0.09	0.09	− 0.09	0.03	− 0.05	0.05	− 0.08	0.19	0.22
Free testosterone (ng/l)	32	− 0.05	− 0.13	− 0.11	0.10	− 0.15	0.20	0.15	0.11	0.04	0.21	0.09
SHBG (nmol/l)	32	− 0.02	0.05	0.09	− 0.14	0.05	− 0.22	− 0.19	− 0.09	− 0.09	− 0.19	− 0.01
DHEA-S (μmol/l)	32	− 0.06	0.01	− 0.15	0.21	− 0.09	0.29	0.28	0.22	0.10	0.01	− 0.06
PLI	55	− 0.13	0.27^2^	0.03	0.20	0.33^3^	0.33^4^	0.29^6^	0.13	0.19	− 0.12	0.23
Fasting glucose (mg/dl)	32	0.12	− 0.05	− 0.01	0.04	− 0.16	− 0.03	0.06	0.05	− 0.01	− 0.17	− 0.24
Fasting insulin (μU/ml)	32	0.18	− 0.245	− 0.14	− 0.18	− 0.23	0.02	− 0.27	− 0.12	0.08	0.09	0.24
HOMA-IR	32	0.13	− 0.04	− 0.13	0.09	− 0.09	0.13	− 0.08	− 0.03	0.15	0.25	0.13
TC (mg/dl)	32	− 0.01	− 0.05	0.00	− 0.08	0.04	− 0.18	− 0.32	− 0.12	− 0.07	− 0.06	0.19
LDL-C (mg/dl)	32	0.05	0.06	− 0.01	0.07	0.16	− 0.08	− 0.28	− 0.01	0.04	− 0.05	0.26
HDL-C (mg/dl)	32	− 0.39^1^	0.16	0.17	0.13	0.12	− 0.36^5^	− 0.19	− 0.26	− 0.30	− 0.09	− 0.24
TG (mg/dl)	32	0.30	− 0.24	− 0.09	− 0.18	− 0.30	0.01	− 0.03	0.00	− 0.11	0.01	0.28

*BMI* body mass index, *PLI* plaque index, *GI* gingival index, *BOP%* bleeding on probing score, *PD* probing depth, *FSH* follicle-stimulating hormone, *LH* luteinizing hormone, *SHGB* sex hormone–binding globulin, *DHEA-S* dehydroepiandrosterone sulfate, *HOMA-IR* homeostatic model assessment *of* insulin resistance, *TC* total cholesterol, *HDL-C* high-density lipoprotein cholesterol, *LDL-C* low-density lipoprotein cholesterol, *TG* triglycerides

Statistically significant correlations: ^1^*p* = 0.0283, ^2^*p* = 0.0497, ^3^*p* = 0.0154, ^4^*p* = 0.0145, ^5^*p* = 0.0452, ^6^*p* = 0.0380, ^7^*p* = 0.0478

## Discussion

The association between gingivitis and hormonal changes during puberty, pregnancy, menstrual cycles, and following medication with first-generation oral contraceptives is a widely known phenomenon [1]. Sex steroid hormones affect periodontal structures through interactions with specific periodontal and immune cells, altering periodontal vasculature and affecting the composition of subgingival microflora [24, 25].

Several mechanisms have been proposed to explain the action of sex steroids on oral bacteria. Some bacteria such as *P. intermedia* and *P. gingivalis* have been shown to possess the ability to substitute estrogen and progesterone for vitamin K compounds as electron carriers in bacterial anaerobic respiration [26]. It may explain the association between increased estrogen and progesterone concentrations and the elevated counts of *P. intermedia* and *P. gingivalis* during pregnancy [27–29]. It is thought that testosterone and its metabolites may also contribute to bacterial growth; however, there is little evidence in the literature to support this claim [30].

Excessive androgen production is a significant trait of PCOS [10, 11]. Besides, the overproduction of luteinizing hormone is evident, and the absence of the peak level of the hormone results in increased secretion of estrogen and progesterone [22]. According to Akcali et al. [5], such hormonal changes are likely to influence the levels of periodontal pathogens. Their findings demonstrated that the salivary levels of *P. gingivalis*, *F. nucleatum*, *S. oralis*, and *T. forsythia* were higher in subjects with PCOS suffering from gingivitis than in matched systemically healthy women. *A. actinomycetemcomitans* and *T. denticola* levels were similar in both study groups. The authors observed no difference between periodontally healthy women with PCOS and systematically healthy women with and without gingivitis. The results of Lindheim et al.’s study [12] showed that PCOS was associated with a decreased abundance of salivary Actinobacteria, common members of the oral microbiota that have been reported to be reduced in periodontal disease. They suggested that the reduction in the relative abundance of Actinobacteria in patients with PCOS may further provide a more favorable environment for pathology-associated bacteria, which can result in periodontal disease in the presence of other permissive factors. No statistically significant differences were detected between the counts of most of the other pathogens in the study and the control group. Hormonal and metabolic components of the syndrome were not associated with salivary microbiome parameters.

Our study did not reveal adverse microbiome alteration in patients with PCOS. On the contrary, the composition of subgingival plaque seemed to be in favor of patients with endocrinopathy: the control group had a significantly higher level of *P. micros* and a significantly greater percentage of subjects infected by *T. denticola*, *P. micros*, and orange complex bacteria. Recent studies of Al-Hebshi et al. [31] indicate that *P. micros* showed the strongest association with periodontal disease as compared with other known periopathogens. The role of *T. denticola* in the periodontal disease has been widely confirmed over two decades ago. Its abundance is highly correlated with periodontal pocket depth, increasing gingival exudate, bleeding on probing, and the loss of connective tissue attachment [32]. Would it mean that young patients with PCOS are less susceptible to colonization by periopathogens as compared with healthy controls? On the other hand, GI and BOP% indices were similar in both groups. Thus, one could speculate that PCOS is associated with an increased risk of gingival inflammation in the presence of relatively lower amounts of periopathogens. It must be remembered that the number of the examined patients was low, and further studies are needed to verify the obtained results. A simple alternative explanation for the observed differences between groups is that they reflect the natural diversity of the oral microflora found in the general population [33].

Interestingly, the counts of periopathogens did not correlate significantly with GI and PD. These results argue with the other published studies that report a positive correlation between gingival inflammation, pockets depths, and bacterial levels [23, 31, 32, 34]. The possible explanation of this phenomenon might be attributed to the age of examined patients and their oral health maintenance. Most of them presented good oral hygiene, none had abnormal PD, and only 6 developed moderate gingivitis. BOP%, which has been recently proposed as an adequate index for the diagnosis of gingivitis [2], was significantly correlated with the number of bacteria from the red complex. Separate statistical analysis carried out after the selection of two subgroups of patients with different severity of gingivitis confirmed that moderate gingival inflammation was associated with the presence of higher levels of all species belonging to red complex and a higher level of *P. micros*.

The bacterial complexes reflect microbial succession in developing subgingival and supragingival dental biofilms and gingivitis [32, 35–37]. Early colonizers include members of the green, yellow, and purple complexes. In the present study, two early colonizers were tested: *C. gingivalis* (green complex bacteria) and *A. actinomycetemcomitans* (pathogen responsible for aggressive forms of periodontitis). The orange complex bacteria appear after early colonizers and include several anaerobic gram-negative species such as *P. intermedia*, *P. micros*, and *F. nucleatum*. The red complex, which appears later during biofilm development, comprises three periodontal pathogens, namely, *P. gingivalis*, *T. denticola*, and *T*. *forsythia*. Red complex presents a portion of the climax community in the biofilms at sites expressing established gingivitis or progressing periodontitis [33]. Among the 9 periodontal pathogens tested, *C. gingivalis*, *P. intermedia*, and *P. gingivalis* were the most abundant species, while the least abundant was *A. actinomycetemcomitans*. Regarding frequency of the occurrence, *C. gingivalis* was isolated from all subjects, followed by *P. micros*, and then *T. denticola*, while *E. nodatum* from the orange-associated complex was not detected in any of the subjects. The results are comparable with those obtained by Mitova et al. [38] who examined subgingival microflora of children aged 10–14. In this study, *C. gingivalis* was the most abundant pathogen while *A. actinomycetemcomitans* and *E. nodatum* were very rare.

It is difficult to reach a full comparison of our results with those of other authors due to the variability in age, sex, and oral health status of the examined individuals and different sampling methods. However, based on the literature, the abundance of green complex bacteria seems to be associated with periodontal health, while bacteria from orange and red complexes are detected at elevated levels in diseased subgingival sites [35–38]. It is with no surprise that young participants of our study, the majority of whom presented good oral hygiene, had high levels of green complex bacteria and species typical of steroid hormones–induced gingivitis (*P. intermedia* and *P. gingivalis*) [27–29].

In individuals affected by PCOS, there were also some significant correlations between the abundance of some species, hormonal parameters, and lipid profile. The count of *C. gingivalis*, a common species from the green complex, was positively correlated with the level of estradiol. *C. gingivalis* participates in various types of infections, including periodontal disease and acute respiratory infections, the severity of which depends on the patient’s immune status [39]. Although this bacterium does not utilize sex hormones, it was frequently detected in children with pubertal gingivitis, together with *P. intermedia* [24, 38]. The abundance of *A. actinomycetemcomitans* and orange complex bacteria was inversely related to HDL-C level. Orange complex bacteria, including *F. nucleatum*, *P. micros*, and *P. intermedia*, are the secondary colonizers of the subgingival plaque. *A. actinomycetemcomitans* is considered an essential microbial factor in aggressive periodontitis, usually with early-onset and combined with various defects in the immune response [33, 38]. It has been suggested that periodontal disease and the presence of periopathogens may influence lipid metabolism, inducing changes in the concentration of cytokines [40]. Recently Lee et al. [41] proved that the prevalence of gingivitis in adolescents is positively correlated with low HDL-C level. Jaramillo et al. [42] noted that periodontitis was associated with distorted lipid profiles and that the serum levels of *P. gingivalis* and *A. actinomycetemcomitans* antibodies were a risk factor for decreased HDL-C levels. Choi et al. [43] provided evidence suggesting that the periodontal inflammation induced by exposure to major periodontal bacteria (*P. gingivalis*, *T. denticola*, *T. forsythia*, and *P. intermedia*) induced dyslipidemia by lowering serum HDL-C level. On the other hand, lipid metabolism anomaly in the blood may also affect periodontal disease progress. Animal studies have shown that hyperlipidemia can predispose the host to oral infection, by impairing proper immune response to bacteria challenge [44, 45].

Apart from abnormal lipid profiles, other parameters of metabolic syndrome, such as insulin resistance, have been linked to oral microflora composition. Recent study by Shrestha et al. [46] showed that specific bacterial groups, including *P. intermedia*, *P. gingivalis*, *T. denticola*, *T. forsythia*, were associated with elevated plasma glucose levels in adults. Similarly, Demmer et al. [47] noted that higher colonization levels of *A. actinomycetemcomitans*, *P. gingivalis*, *T. denticola*, and *T. forsythia* are associated with higher prediabetes prevalence among diabetes-free adults. Our study did not reveal a significant association between bacteria counts, fasting glucose, fasting insulin levels, and the index of insulin resistance HOMA-IR. However, our study population was younger than those in above-mentioned studies and did not present abnormal levels of glucose.

Finally, there was a significant correlation between PLI and the number of bacteria from the red and from the orange complex, as well as with the counts of two species: *T. denticola* and *F. nucleatum*. As observed by Nogueira Moreira et al. [48], supragingival plaque control reduced a number of potentially pathogenic subgingival microbiota.

Oral hygiene level was also significantly correlated with both indices of gingival inflammation, which confirms the role of dental biofilm in the etiology of gingivitis [2]. It is a well-known phenomenon, although the magnitude of correlation might be lower in young subjects with healthy gingiva and high oral hygiene standards [49].

The study has several limitations that should be considered during interpretation of the results. Firstly, the number of subjects was low, and the control group was selected without a blood test and ultrasound examination. We tried to address this limitation, referring control subjects to the gynecologist for a non-invasive evaluation. Secondly, the diagnosis of PCOS was based on the original Rotterdam guidelines [11], while some experts recommend the use of more strict criteria during adolescence, when the symptoms of normal puberty may imitate those typical of PCOS [50]. To reduce the possibility of overdiagnosis, we choose subjects who were at least 2 years after menarche and were referred to the hospital due to persistent problems with the menstrual cycle and clinical signs of hyperandrogenism [51]. Thirdly, we did not radiographically assess alveolar bone loss and assess the condition of periodontium based on GI, BOP%, and PD. Finally, the oral microbiome can be affected by the diet [52]. We did not analyze this association, although the wide range of BMI noted in the subjects of the study may express differences in their attitude to nutrition.

On the other hand, this the first study which assesses the presence of periopathogens in young patients suffering from hyperandrogenism. Contrary to our expectations, endocrinopathy was not associated with higher pathogenicity of subgingival biofilms.

## Conclusions

Adolescent patients with PCOS did not differ significantly from healthy controls with respect to oral hygiene, periodontal health, and the total bacteria count. The study did not reveal adverse microbiome alteration in patients with PCOS.

Interestingly, the levels of estradiol and HDL-C were correlated with microbiological parameters. It suggests that there is an association between the composition of subgingival microflora, endocrine, and metabolic components of PCOS. The observed correlations were weak which indicates that the majority of between-subject variation in oral microbiome profiles remains to be explained.

Further studies are needed to confirm these preliminary results and understand the cause relationship between hormonal and metabolic abnormalities, subgingival microflora, and oral health in patients with PCOS.
